# Effect of epithelium ATP release on cyclic pressure-induced airway mucus secretion

**DOI:** 10.1042/BSR20130109

**Published:** 2014-01-14

**Authors:** Jin Tong, Xiang-dong Zhou, Juliy M. Perelman, Victor P. Kolosov

**Affiliations:** *Department of Respiratory Medicine, the Second Affiliated Hospital, Chongqing Medical University, Chongqing 400010, China; †Far Eastern Scientific Centre of Physiology and Pathology of Respiration, Russian Academy of Medical Sciences, Blagoveshchensk 675000, Russia

**Keywords:** ATP, calcium, cyclic pressure, mucins, 16HBE, human bronchial epithelial cells, AM, acetoxymethyl ester, ASL, airway surface liquid, BAPTA, 1,2-bis-(*o*-aminophenoxy)ethane-*N*,*N*,*N*',*N*'-tetra-acetic acid, GAPDH, glyceraldehyde 3-phosphate dehydrogenase, HRP, horseradish peroxidase, MTT, 3-(4,5-dimethylthiazol-2-yl)-2,5-diphenyl-2*H*-tetrazolium bromide, MUC, mucin, RB-2, reactive blue-2, RT–PCR, reverse transcription–PCR

## Abstract

The cyclic mechanical effect of airflow during breathing creates the optimal airway hydration state. MUC (mucin) 5AC is an important component of the airway mucus. The formation of MUC5AC is related to ATP and intracellular calcium in the epithelial cells. In this study, we evaluated the effect of ATP release from intracellular calcium in epithelial cells on cyclic pressure-induced mucus secretion in the airway. 16HBE (human bronchial epithelial cells) were cultured *in vitro* on cyclically tilted cultured plates and divided into five groups: control, tilt, tilt and BAPTA–AM (1,2-bis-(*o*-aminophenoxy)ethane-*N*,*N*,*N*',*N*'-tetra-acetic acid–acetoxymethyl ester), tilt and EGTA and tilt and RB-2 (reactive blue-2). The shear stress and compressive stress were induced by the surface tension of the liquid, atmospheric pressure and liquid gravity. Cell activity, MUC5AC mRNA expression level, MUC5AC protein expression level and ATP release and intracellular calcium changes were measured with the MTT (3-(4,5-dimethylthiazol-2-yl)-2,5-diphenyl-2*H*-tetrazolium bromide) assay, RT–PCR (reverse transcription–PCR), HPLC and inverted fluorescence microscope, respectively. We detected that cyclic pressure significantly increased MUC5AC secretion and ATP release. The enhanced ATP release could be inhibited by both BAPTA–AM and RB-2, while EGTA did not have a suppressive effect. BAPTA–AM, EGTA and RB-2 did not obviously inhibit MUC5AC mRNA expression. Cyclic pressure did not induce MUC5AC secretion in the airway mucus epithelium via Ca^2+^-dependent ATP release, and nearly all Ca^2+^ was provided by stored intracellular Ca^2+^.

## INTRODUCTION

Airway mucus is a heterogeneous mixture of secreted polypeptides, cells and cellular debris that is present in the airway surface lining fluid subphase or tethered at the fluid surface by oligomeric MUC (mucin) complexes [1,2].

The airway luminal surface is coated by a thin layer of ASL (airway surface liquid), which is composed of water, MUC, lysozyme, lactoferrin and various polypeptides. MUC imparts viscoelasticity to mucus and participates in foreign particle adhesion and pathogen binding, prompting their clearance via ciliary beating or coughing. MUC5AC is one of the most prominent secreted MUCs in the respiratory tract. Under normal respiratory rhythm and mucus production and secretion, the absorption of water and a variety of ions create a physiological balance, known as the steady state [3]. At present, some studies have confirmed that the cyclic mechanical stimulation generated by airflow and transmural pressures during breathing could establish optimal ASL volume and airway hydration by prompting ATP release and activating purinoceptors located in the apical membrane of airway epithelial cells, thus regulating the secretion and absorption of water and various ions [4,5]. However, the exact effects of cyclic compressive pressure on the production and secretion of MUC remain unclear. In the present study, we used 16HBE (human bronchial epithelial cells) to understand the effects of cyclic compressive pressure.

## MATERIALS AND METHODS

### Materials and reagents

The following materials and reagents were used: 16HBE (A.T.C.C.); RPMI-1640 culture medium; TRIzol bovine serum (Hyclone); standard ATP powder (Sigma); an intracellular selective Ca^2+^ chelator (BAPTA-AM (1,2-bis-(*o*-aminophenoxy)ethane-*N*,*N*,*N*′,*N*′-tetra-acetic acid–acetoxymethyl ester)) (Sigma); the Ca^2+^ chelator EGTA (Sigma); RB-2 (reactive blue-2), which blocks purinoceptor P2Y (Sigma); an HRP (horseradish peroxidase)-conjugated goat anti-mouse IgG (Sigma); TRIzol solution for RNA extraction; an RT–PCR (reverse transcription–PCR) testing kit (Invitrogen); the MU5AC monoclonal antibody (Santa Cruz); type-I collagen (Sigma); an ELISA testing kit; an HP1100 high performance liquid chromatograph; a weak anion chromatography column (BD); the Ca^2+^ fluorescence indicator Fluo-3-AM (Beyotime Institute of Biotechnology); an inverted fluorescence microscope (Olympus); 6- and 96-well silica gel culture plates (Flexcell International); an electric swinging plate (customized for rocking equipment; the duration and frequency of swinging is also controllable) (Biotechnology Company). The remaining reagents were domestic and analytical grade (Zhongshan Goldenbridge Biotechnology).

### Cell culture and processing [6]

Isolated 16HBE cells were seeded on 6-well elastic membranous silica gel culture plates precoated with type-I collagen (5 × 10^5^ cells per well). Cultures were incubated in a tissue culture incubator [5% (v/v) CO_2_] at 37°C. Cells were washed routinely. When the fusion rate was approximately 70–80%, the cells were washed twice with PBS and randomly divided into the following groups. (1) Control group: cells were untreated and not exposed to tilt. (2) Tilt group: cells were cultured with tilt in fresh serum-free RPMI-1640 medium. (3) Tilt and BAPTA–AM group: cells were cultured in fresh serum-free RPMI-1640 medium supplemented with 0.1 mmol/l BAPTA–AM to chelate intracellular Ca^2+^. BAPTA–AM easily penetrates the cell membrane, but BAPTA is unable to enter the cell. Within the cell, BAPTA can separate from AM and chelate intracellular Ca^2+^; thus, the compound cannot affect extracellular Ca^2+^. (4) Tilt and EGTA group: cells were pretreated with 0.12 mmol/l EGTA, which cannot penetrate the membrane, for 30 min to chelate extracellular Ca^2+^. (5) Tilt and RB-2 group: cells were pretreated with 0.2 μmol/l RB-2 for 30 min. RB-2 blocks purinoceptor P2Y and can interrupt ATP release.

### Cyclic pressure apparatus [7,8]

**Figure 1:**
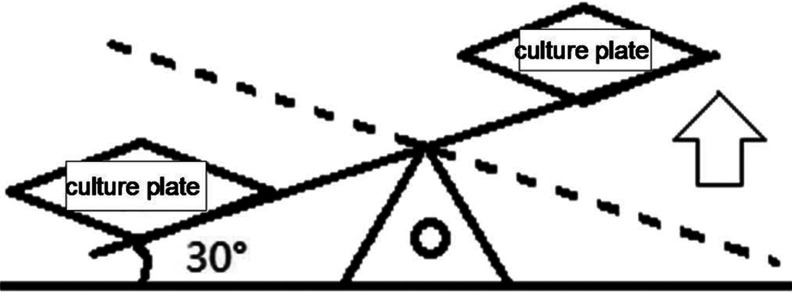
Motor-operated seesaw

**Figure 2:**
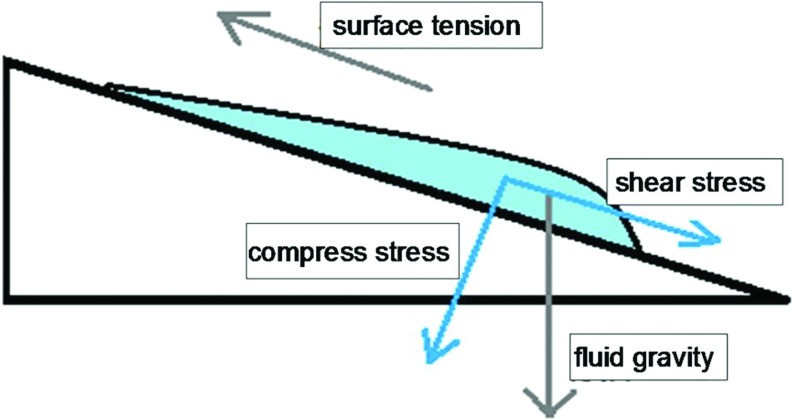
Gravity and surface tension of fluid

All the culture plates except the control group were placed on a motor-driven rocking board. A schematic diagram of the instrumentation is shown in Figure 1. In this system, the board is tilted to 30°, and the culture medium flows towards the lower side due to gravity. Thus, an extremely thin layer of culture medium adhered to the upper side of the cells, and some cells were exposed to air, similar to air–liquid conditions. Cells on the upper side were stretched by the surface tension created by these conditions; cells on the lower side were compressed by the weight of the culture medium. We resolved the fluid gravity and surface tension in parallel and perpendicularly into two components along the culture plate, with each component indicating the influence of the shear and compressive stress of airflow on the airway epithelium. The culture medium, which is comparable with the mucus surface tension under normal conditions, had a pressure of approximately 2 cm H_2_O on the lowest cells (see Figure 2). The motor-driven rocking board swung gently every 15 min at an angle of 30° relative to the ground, attempting to avoid the influence of motion itself on cells. Culture plates were alternately and tautologically tilted right and left every 15 s over 30 min. The control culture plates were placed in an incubator without motion for the same duration of time. Culture medium from every group was collected and assayed for the ATP concentration. This procedure was repeated after each change of the culture medium over six changes.

### MTT [3-(4,5-dimethylthiazol-2-yl)-2,5-diphenyl-2*H*-tetrazolium bromide] reduction assay of cell activity

In total, 200 μl of a cell suspension with a cell density of 1×10^4^ per ml was introduced into each well of a 96-well plate for 24 h. Then, serum-free culture medium was replaced for another 24-h culture period. An MTT assay was performed at 10 s, 15 s, 30 s and 45 s across the 30° tilts (i.e., approximately one-third of cells were exposed to air without culture medium). Twenty-four h later, 1 mmol/l BAPTA–AM, 20 μmol/l RB-2 and 0.12 mmol/l EGTA were separately introduced into the culture wells for the MTT assay.

The absorbance (*A*) value of each well was measured at a wavelength of 570 nm with the enzyme-labelled instrument. The control well was measured in parallel without any drug introduction; the blank control only contained the culture medium. The survival rate of cells (100%) was estimated by the average value of the six replicate wells of each experimental group as follows:

$$\left(A_{\mathrm{experiment}}-A_{\mathrm{blank}--\mathrm{control}}\right)/\left(A_{\mathrm{control}}-A_{\mathrm{blank}--\mathrm{control}}\right)\times 100\%,$$

where *A*_experiment,_ *A*_control_ and *A*_blank–control_ represent the *A* value of the experimental, control, and blank control groups, respectively.

### Quantification of secreted MUC5AC with ELISA

The 96-well ELISA plates with 50 μl of supernatant were incubated at 4°C overnight and blocked for 1 h with bovine serum at room temperature. Then, the plates were incubated with 45 ml mouse anti-MUC5AC mAb (diluted 1:100 in 50 μl PBS with 0.05% Tween-20), followed by 100 μl of goat anti-mouse-HRP (1:10000 dilution) for 1 h. MUC5AC levels were determined with tetramethyl benzidine staining and *A* at 450 nm and were assessed relative to the standard concentration.

### Measurement of MUC5AC mRNA by RT–PCR

The total RNA was extracted from each group with TRIzol and stored at −20°C. Samples were assayed with 1% (w/v) agarose gel electrophoresis to assess *A* at 260–280 nm (the *A* ratios at 450–595 nm were between 1.8 and 2.0). Then, the *A* ratios were preliminarily quantified according to the *A* value at 260 nm. The two-stage method was used for RT–PCR. First-strand cDNA was obtained via a synthesis kit according to the manufacturer's instructions. The upstream PCR primer sequence was 5′-CTGCCAAGTGGTCAGAGGG-3′, and the downstream was primer sequence was 5′-TGTCCAGGAAGGTGTAGTAGGTG-3′. GAPDH (glyceraldehyde 3-phosphate dehydrogenase) was chosen as the endogenous control gene, with upstream and downstream primer sequences of 5′-GGGAAGGTGAAGGTGGGA-GTG-3′ and 5′-AGCAGAGGGGGCAGAGATGAT-3′, respectively. In total, 4.0 μl MgCl_2_, 5.0 μl 10×PCR buffer solution, 1.0 μl upstream primers, 1.0 μl downstream primers, 4.0 μl dNTP, 2.0 μl cDNA and 0.5 μl Taq enzyme (5 units/μl) were combined in sterilized Eppendorf tubes and mixed well in a final volume of 50.0 μl. PCR amplification was performed as follows after an adequate centrifugation of this mixture: denaturing at 94°C for 3 min, followed by 35 repeated cycles at 94°C for 45 s, 54°C for 30 s and 70°C for 60 s. An extension step was performed at 72°C for 10 min to complement the DNA template strand. Finally, the PCR products were assayed with 2% agarose gel electrophoresis, and the amplified bands were analysed with grey-scale scanning via a Bio-Rad gel imaging analysis system to determine the relative amount of mRNA of each target gene compared with the relative amount of the housekeeping gene GAPDH.

### Level of ATP in the culture medium as measured by HPLC

The buffering agents NaH_2_PO_4_ and KH_2_PO_4_ were used at concentration of 0.25 mmol/l to maintain a pH of 7.0 during the mobile phase of the weak ion-exchange column chromatography. The mixture descended the column at a speed of 1.5 ml/min and could be detected postcolumn with a wavelength detector at 259 nm. A standard ATP solution was prepared as an external standard; a standard ATP sample of 1.00 mg was dissolved in double-distilled water at a final volume of 5.00 ml and concentration of 200.00 μg/ml. ATP levels in the culture medium were calculated as follows:

Sample_CON_=(Sample_PEAK−AREA_/Standard_PEAK−AREA_)×Standard_CON_,

where the Sample_CON,_ Standard_CON,_ Sample_PEAK−AREA_ and Standard_PEAK−AREA_ indicate the concentration and peak area of sample solution and external standard, respectively.

### Determination of the intracellular Ca^2+^ concentration with inverted fluorescence microscopy

The upper culture medium was replaced with 10 μmol/l fluo-3/AM after culture termination. Following incubation for 30 min, the cells were washed with a balanced salt solution three times to eliminate the extracellular fluorescent agent. Level medium was placed on the stage of an inverted fluorescence microscope to observe the fluctuations in the intracellular calcium concentration. Single unit recording was used for calcium fluorescence imaging, and time sequence software (Meridran) was used to calculate fluorescence intensity and calcium concentrations. Maximum intensity was defined as the peak intracellular calcium concentration.

### Statistical analysis

Comparisons between different groups were performed with the SPSS 17.0 software program, and results are displayed as the means±S.E. (*x*±s). A parametric or non-parametric test was performed based on the homoscedasticity or heteroscedasticity of the data, respectively; ANOVA was used for homogeneous data; and a rank sum test was for used for heterogeneous data. An LSD (least significant difference) test was conducted when the differences between several groups were significant for multiple comparisons. *P*<0.05 was considered statistically significant.

## RESULTS

### Measurement of cell activity

When the cultured board tilted to 30°, the cellular survival rates at 0, 10, 15, 30 and 45 s were 100.0±0.2, 99.5±0.1, 99.2±0.3, 98.6±0.5 and 97.4±0.1%, respectively. When 1 mmol/l BAPTA–AM, 20 μmol/l RB-2 and 0.12 mmol/l EGTA were added to the cultured cells, the survival rates were 98.5±0.2%, 99.1±0.3%, 98.0±0.2%, respectively, indicating that tilting the cultured board within 30 s (i.e., partial exposure to the air no longer than 30 s) had no significant negative effect on the cells. Thus, in the present experiment, we tilted each side of the cultured board for 15 s. Additionally, the applied concentration of BAPTA–AM, RB-2 and EGTA did not injure the cells.

### Influence of cyclic pressure and the addition of BAPTA–AM, EGTA and RB-2 on mRNA and MUC5AC protein levels

**Figure 3:**
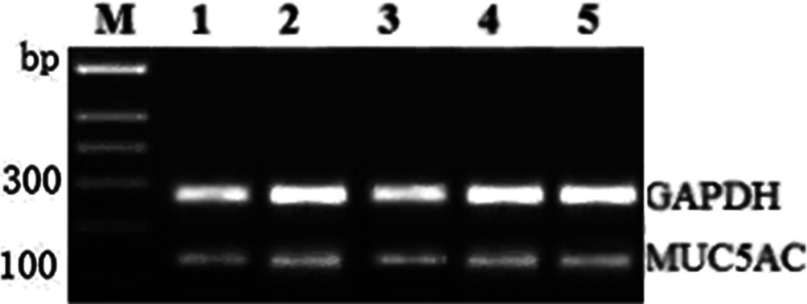
RT–PCR analysis of MUC5AC mRNA expression M: DNA marker; 1. control group; 2. tilt group; 3. tilt and BAPTA–AM group; 4. tilt and EGTA group; and 5. tilt and RB-2 group. GAPDH was used as a control.

The MUC5AC mRNA expression level in the tilt group cells, which were exposed to cyclic pressure, changed to 5.8±0.01 and was obviously different from that of the control 5.4±0.01(*t*=−6.40, *P*=0.010). Furthermore, the MUC5AC mRNA expression level in the tilt group was no significant different to that of the tilt groups that were treated with BAPTA–AM, EGTA and RB-2 with relative values of 5.6±0.02(*t*=−2.10, *P*=0.081), 5.9±0.01(*t*=1.40, *P*=0.192) and6.0±0.01(*t*=2.50, *P*=0.063), respectively, as shown in Figure 3.

### Effect of cyclic pressure wave on ATP release and MUC5AC secretion in 16HBE cells

**Figure 4:**
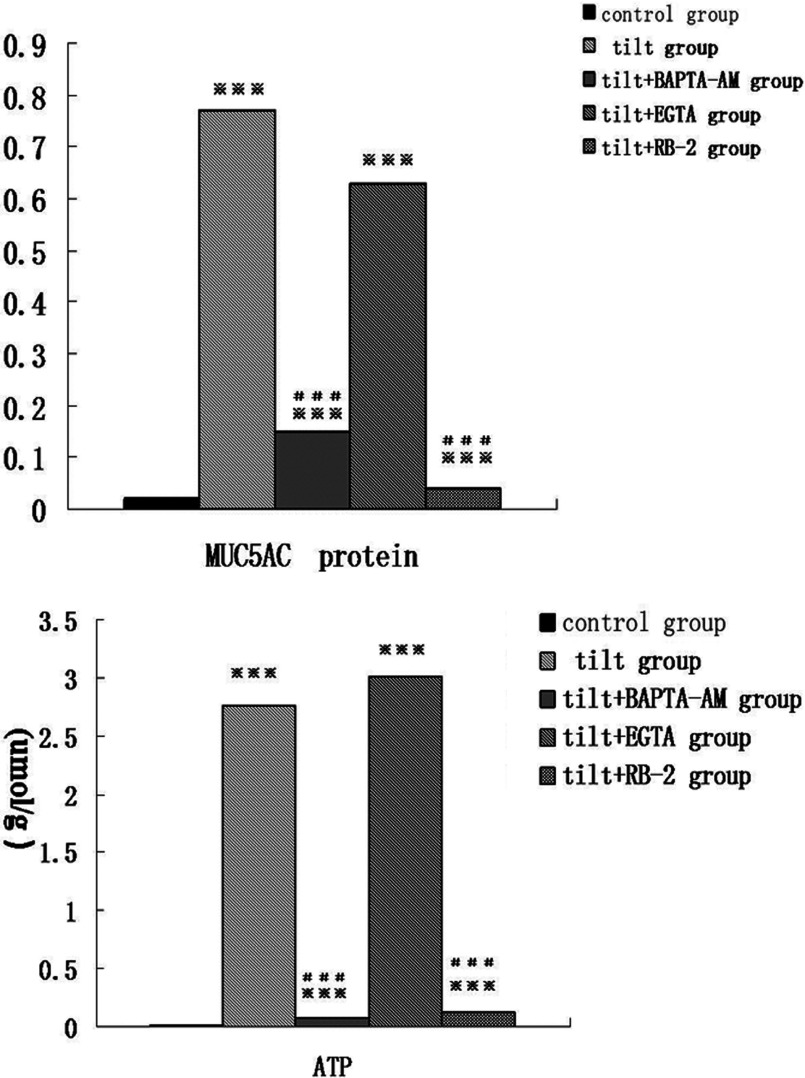
Effects of mechanical ventilation on MUC5AC secretion and ATP release in 16HBE cells (A) Effects of mechanical ventilation on MUC5AC secretion in 16HBE cells. (B) Effects of mechanical ventilation on ATP release in 16HBE cells. ***P<0.01 versus control group; ^###^*P*<0.01 versus tilt group. Data were analysed by ANOVA with SNK *post-hoc* analysis. The data are presented as the means±S.D. (*n*=6).

The amount of ATP released and MUC5AC secreted from the tilt group cells after stimulation with cyclic pressure were 2.76±0.47 μmol/g and 0.77±0.26, respectively, and were 0.00±0.01 μmol/g and 0.02±0.01 in the control group, respectively, indicating a significant increase for both ATP release (*t*=12.07, *P*=0.008) and MUC5AC secretion (*t*=−46.44, *P*=0.000) with cyclic pressure. Further, both BAPTA–AM(0.08±0.01 μmol/g, 0.15±0.04), a calcium chelator and the selective P2Y-receptor antagonist RB-2(0.12±0.45 μmol/g, 0.04±0.07) significantly inhibited the pressure-induced increase in release and secretion. The inhibitory role of EGTA (3.01±0.07 μmol/g, 0.63±0.03) was not significant (see Figure 4).

### Intracellular calcium concentration as measured with an inverted fluorescence microscope

**Figure 5:**
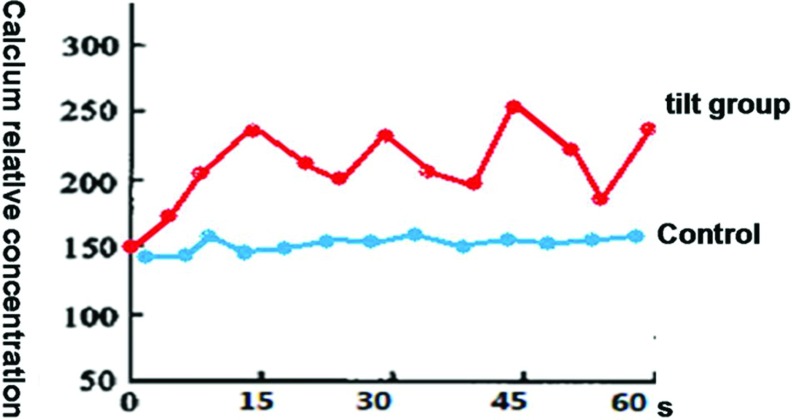
Concentration–time curve of intracellular calcium in the control and tilt groups The data was relative concentration, which were the groups under different condition compared with the control.

**Figure 6:**
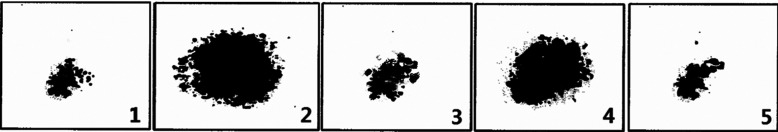
Fluorescence intensity of intracellular calcium in a single cell as measured with an inverted fluorescence microscope The maximum fluorescence intensity indicated the peak calcium concentration. Single unit recording was selected for calcium fluorescence imaging, and time sequence software was adopted to calculate fluorescence intensity and calcium concentration. 1. control group; 2. tilt group; 3. tilt and BAPTA–AM group; 4. tilt and EGTA group; and 5. tilt and RB-2 group.

The fluorescence intensity corresponding to the intracellular calcium concentration in the control group was stable and remained unchanged over time. In the tilt group, the fluorescence intensity peaked when the swing tilted to the maximal angle, followed by a gradual decrease as the angle was reduced and a rapid increase when the angle was increased. Such fluorescence intensity fluctuation repeated with every swing cycle and remained consistent with the swing period of 15 s (see Figure 5). The intracellular calcium concentration of the tilt group was significantly higher than that of the control and declined after pretreatment with BAPTA–AM and RB-2; however, no changes were observed with pretreatment with EGTA (see Figure 6).

## DISCUSSION

Tidal breathing is one requirement for lung defence. Between tidal breathing, the link of ASL and the rates of mucociliary clearance are raised. The thin layer of ASL lining the airway surface is the first line of defence against bacterial infection [9].

During normal rhythmic breathing, the absorption and transport of water and ions, the production and secretion of MUC, and ciliary beating are well balanced [10]. Two types of respiration mechanical forces dominate in the airways: airflow across the surface of the airway epithelium produces wall shear stress and a trans-airway gradient pressure, imparting stretch and compress forces on the surface epithelium [11,12]. In the present experiment, we utilized surface tension and gravity by tilting a cultured plate to simulate shear stress and compressive stress. Our *in vivo* experiment successfully mimicked the stretch and compression forces applied on the human airway epithelium during normal breathing, and the duration and frequency of the force was precisely regulated at a frequency consistent with normal tidal breathing, to well-differentiated 16HBE cell cultures. Using the model system of phasic airway compression, we established the relationship between the cyclic compressive stress and steady-state ASL. Further, factors that were irrelevant to this study were excluded from the analysis.

In previous study, a lot of findings proved that the hydration state of the ASL is affected by regulating ENaC (epithelial Na^+^ channel)-mediated Na^+^ absorption and CaCC-mediated Cl^−^ secretion [13]. The relationship of Ca^2+^ absorption and the state of ASL was not clear. MUC5AC is the important constituent of ASL. In the study, we researched that whether the secretion of MUC5AC and the releasing of ATP were affected by regulating intracellular or extracellular Ca^2+^ absorption.

Our data showed that cyclic pressure induced the secretion of ATP and MUC5AC in the airway epithelium and increased the intracellular calcium concentration. This promotion could be inhibited after treatment with the P2Y receptor antagonist RB-2. BAPTA–AM, a selective intracellular calcium chelator, significantly blocked ATP release. Another calcium chelator, EGTA, which is not cell permeant, was used to control the level of extracellular Ca^2+^ and did not affect ATP release in cells under stimulation of cyclic pressure. Additionally, our results indicated that ATP is among the many factors that increase MUC5AC protein secretion but not mRNA expression. Intracellular calcium played a crucial role in this process, and the inflow of extracellular calcium was absent.

In summary, we confirmed that cyclic pressure regulates mucus secretion in the airway epithelium through Ca^2+^-dependent ATP release and that only intracellular Ca^2+^ functions in this process.

MUC5AC secretion would affect the ASL hydration state. This 7-μm layer represents the fully hydrated ASL environment in which the cilia are able to fully extend and beat [14], so it could make the mucus clearance perfect. The balance regulation of intracellular Ca^2+^ could maintain a normal secretion of MUC5AC and perfect volume ASL, which make airway epithelium ideal defensive state. Based on our primary results, future studies should explore the precise pathways and mechanisms involved.
