# Expression of Concern: Effect of epithelium ATP release on cyclic pressure-induced airway mucus secretion

**DOI:** 10.1042/BSR-20130109_EOC

**Published:** 2021-04-07

**Keywords:** ATP, calcium, cyclic pressure, mucins

The Editorial Office has been made aware of potential issues surrounding the scientific validity of this paper, hence has issued an expression of concern to notify readers whilst the Editorial Office investigates.

